# Advanced Interrogation of Fiber-Optic Bragg Grating and Fabry-Perot Sensors with KLT Analysis

**DOI:** 10.3390/s151127470

**Published:** 2015-10-29

**Authors:** Daniele Tosi

**Affiliations:** Nazarbayev University, School of Engineering, 010000 Astana, Kazakhstan; E-Mail: daniele.tosi@nu.edu.kz; Tel.: +7-7172-70-5855

**Keywords:** optical fiber sensors (OFS), fiber Bragg grating (FBG), Fabry-Perot interferometry (FPI), optical signal processing, Karhunen-Loeve Transform (KLT), optical sensor demodulation

## Abstract

The Karhunen-Loeve Transform (KLT) is applied to accurate detection of optical fiber sensors in the spectral domain. By processing an optical spectrum, although coarsely sampled, through the KLT, and subsequently processing the obtained eigenvalues, it is possible to decode a plurality of optical sensor results. The KLT returns higher accuracy than other demodulation techniques, despite coarse sampling, and exhibits higher resilience to noise. Three case studies of KLT-based processing are presented, representing most of the current challenges in optical fiber sensing: (1) demodulation of individual sensors, such as Fiber Bragg Gratings (FBGs) and Fabry-Perot Interferometers (FPIs); (2) demodulation of dual (FBG/FPI) sensors; (3) application of reverse KLT to isolate different sensors operating on the same spectrum. A simulative outline is provided to demonstrate the KLT operation and estimate performance; a brief experimental section is also provided to validate accurate FBG and FPI decoding.

## 1. Introduction

Optical fiber sensors (OFS) are established as a pillar in the sensing technology landscape [1]. Compared to mainstream sensing systems, such as micro-electromechanical systems and wireless sensor networks, OFS possess specific advantages: microscopic size of the sensors and delivery fibers, immunity to electromagnetic interference, biological compatibility, resistance to corrosion and chemical agents, and the possibility to operate over distances ranging from a few millimeters to tens of kilometers along a single fiber. Currently, OFS are well established in the detection of physical and electrical parameters, and growing in the biochemical sensors niche.

From the point of view of the sensing system as a whole, one of the main trends of OFS-based technologies is to operate the spectral detection in the optical spectrum domain. In this framework, the sensor acts as a wavelength-selective element, such as a narrowband filter, a cavity, or an absorption peak; this wavelength pattern introduced by the sensor changes as the measurand is subjected to variations. Thus, the interrogation system detects the spectrum of the sensor, and records its variations.

A large number of optical fiber sensing systems operate with this approach. Fiber Bragg gratings (FBGs) [2,3,4,5,6,7,8] are the most popular type of such sensors. FBGs are reflection filters, with a very narrow bandwidth, and are sensitive to both strain and temperature, with a nearly instantaneous response. The recent advances on FBG fabrication [9,10], as well as the possibility to inscribe several FBGs operating on the same fiber in a wavelength-division multiplexing (WDM) approach [11], are elevating FBGs as an inexpensive, compact, and well performing technology, with several applications in structural health monitoring [5,6], infrastructures [11], oil and gas [7,8], and medical devices [12,13] among others. In addition to uniform FBGs, the literature also features several types of grating-based fiber sensors. These include chirped gratings [14,15], that allow temperature and strain detection on a distributed length; tilted gratings [2,16,17], particularly suited for biochemical sensing; and long-period gratings (LPGs) [18,19], which are transmission gratings with a broadband response. A second class of sensors is represented by Fabry-Perot interferometry (FPI) [20,21,22,23,24,25,26]: FPI sensors are based on a short cavity within the optical fiber, which generates a broadband spectral variation with a periodic pattern. Extrinsic FPI sensors are one of the main architectures for pressure sensing in medical devices [22,23,24], as well as pressure and temperature sensing in pipelines [25,26]. A third class of wavelength-encoded sensors features architectures for biochemical sensors: most notably, fiber-optic sensors based on surface plasmon resonance (SPR) [27], single-multi-single mode (SMS) structures, tapered fibers [28] produce spectral absorption peaks at a specific wavelength.

As FPI and FBGs allow physical and biochemical sensing within a short active length, one of the main trends in OFS is the integration of multiple sensing elements on a single fiber. A hybrid FPI/FBG structure has been first proposed by Bremer *et al*. [29,30], and subsequently expanded for dual temperature and pressure detection [31,32,33]. With the latest improves in sensor fabrication, the evolution of multi-parameter sensing leans to the lab-in-a-fiber platform.

All these typologies of single- and multi-parameter OFS have interrogation based on spectral detection, which significantly differs from other approaches such as interferometry of intensity-based transducing [34,35]. Typical interrogation systems detect the spectrum reflected by the sensing structure on a wavelength interval ranging from 40 nm to 80 nm [36,37,38,39]. Most systems are based on a broadband light source and a spectrometer for detection [36,37]. This approach is straightforward, provides long-term stability, and is relatively inexpensive; but on the other hand, the spectrum is sampled with a coarse wavelength resolution, typically 512 samples over 40 nm or 80 nm interval. The main alternative is represented by an interrogation system based on a scanning laser [38] or a scanning filter [37] and a photodetector: this method provides a denser sampling (1–10 pm) but requires to stabilize and isolate the laser source and isolate, which results in a more expensive system operating in a lower signal to noise ratio (SNR).

A method that estimates spectral shifts of FBG, FPI, and similar sensors is needed in order to estimate the measurand. Over the past decade, several techniques have been proposed for FBG detection. The simplest methods are based on centroid, curve fitting, or other interpolation routines; such routines are now part of several commercial interrogators [36,38]. The spectral correlation method, proposed by Gong *et al*. [40] and Caucheteur *et al*. [41] provides a significant improvement: it is capable of working even in very low SNR conditions, but it is still limited to the resolution of the interrogation system. In 2014, Lamberti *et al*. [42] proposed a method based on fast Fourier transform (FFT): this technique estimates the FBG wavelength shift with 7–35 femtometer accuracy with 10 pm sampling grid.

Similar techniques have also been proposed for FPI sensors [20], in order to measure the variations of the Fabry-Perot cavity. Recent advances have been proposed by Tosi *et al*. [43], with a method based on adaptive filtering. In 2014, Ushakov and Liokumovich analyzed the resolution limits of FPI sensors, based on interrogation detection [44]. The interrogation of dual FBG/FPI sensors has been proposed by Bremer *et al*. [29], using two sources (laser and LED) to separate FBG and FPI spectra. Other approaches separate the spectral portion containing the FBG from the spectrum, and use the remaining part to interrogate the FPI sensor [31].

In this article, the author aims at introducing a new demodulation technique for FBG, FPI, and nearly all class of sensors based on spectral detection. The method is based on Karhunen-Loeve Transform (KLT) [45,46,47] applied to the sensor spectrum. As framed by Maccone [46], the KLT is effective when in decoding the energy of the input signal, and encodes they key information in its high-tank eigenvalues. KLT-based interrogation was introduced in 2015 for the first time in FBG sensing [47]. In this article, the KLT method will be discussed also for FPI sensors, and hybrid FBG/FPI sensors. Then, with suitable variations, the method will be expanded to a plurality of sensors operating on the same spectral range, isolating the individual contributions.

The paper is arranged as follows: Section 2 describes the tracking method based on KLT; Section 3 describes the KLT applied to individual sensors (FBG and FPI); Section 4 describes the KLT applied to hybrid FBG/FPI sensors; in Section 5, a variation of the KLT is proposed to provide interrogation of more complex multi-parameter sensing structures; Section 6 shows some experimental results for FBG and FPI sensing; finally, Section 7 draws conclusions.

## 2. KLT-Based Tracking Method

The detection of FBGs and FPIs is performed with an interrogation device based on a spectrometer. The spectrometer discretizes the optical spectrum on both wavelength and amplitude axes. The spectrum is then regarded as a digital signal *S*[*λ*], sampled over the *N*-size wavelength grid *λ*_1_, *λ*_2_, …, *λ_N_*, with uniform wavelength step δ*λ*. Also the spectral amplitude is sampled on a grid with δ*S* quantization step. The spectrum *S*[*λ*], hereby regarded as a digital signal, can be expressed as the useful spectrum of sensors, with additive noise:

(1) S[λ]=R[λ]+N[λ]

where *R*[*λ*] is the useful spectrum (typically, the reflection spectrum of cascade of sensors) and *N*[*λ*] is noise. The SNR is defined as the ratio between *R* and *N* variances:

(2) $$SNR=\frac{\mathrm{var}\left\{R\left[\lambda \right]\right\}}{\mathrm{var}\left\{N\left[\lambda \right]\right\}}$$

The first step towards estimating wavelength shifts, following Lamberti′s approach [40], is computing the FFT of the spectrum *S*[*λ*]:

(3) $$G\left(f_{1},\ldots ,f_{N}\right)=FFT\left\{S\left[\lambda_{1},\ldots ,\lambda_{N}\right]\right\}$$

where *f*_1_, …, *f_N_* is the normalized frequency. The new digital variable *G*(*f*) is then transformed into its symmetric Toeplitz matrix:

(4) $$\underline{\underline{M}}=\left[\begin{array}{ccccc} G_{1} & G_{2} & G_{3} & \cdots & G_{N}\\ G_{2} & G_{1} & G_{2} & \ddots & \vdots \\ G_{3} & G_{2} & G_{1} & \ddots & G_{3}\\ \vdots & \ddots & \ddots & \ddots & G_{2}\\ G_{N} & \cdots & G_{3} & G_{2} & G_{1} \end{array}\right]$$

where *G_i_ = G*(*f_i_*).

Then, the KLT of the matrix $\underline{\underline{M}}$ is finally performed. The KLT takes the matrix $\underline{\underline{M}}$ as input, and identifies an orthonormal basis $\underline{\underline{V}}$ over which it is represented [45,46]. In this case, a simple version of KLT is implemented, using the singular value decomposition (SVD):

(5) $$\underline{\underline{M}}=\underline{\underline{V}}\times \underline{\underline{D}}\times {\underline{\underline{V}}}^{-1}$$

where $\underline{\underline{D}}$ is a diagonal matrix containing all the eigenvalues of $\underline{\underline{M}}$ on its main diagonal, and $\underline{\underline{V}}$ is its corresponding orthonormal basis that contains on its lines the eigenvectors. Computationally, the SVD is performed using the Cholesky decomposition.

The matrix $\underline{\underline{D}}$ contains the *N* eigenvalues; as $\underline{\underline{M}}$ is symmetric, all its eigenvalues are real numbers. The eigenvalue string is called *ξ* and is sorted in ascending order:

(6) $$\xi =\left|\xi_{1}\right|<\left|\xi_{2}\right|<,\ldots ,<\left|\xi_{N}\right|$$

As noted by Maccone [46], the eigendecomposition is well effective in separating signal from noise. Low-rank eigenvalues, and their correspondent eigenvectors, are mostly affected by noise; conversely, the high-rank eigenvalues confine most of the useful signal energy. As discussed in the next sections, the analysis of the eigenvalue string *ξ* is the core of the optical fiber sensors interrogation.

## 3. Interrogation of FBG and FPI Individual Sensors

### 3.1. Benchmark

A simulation benchmark has been set up to demonstrate the operation of KLT as fiber-optic sensor demodulator, and quantify its performance. In order to reproduce most commercial spectrometers, the wavelength grid is chosen as δλ = 156 pm, over 80 nm range, reproducing 9-bit sampling; the amplitude grid is δ*S* = 1/65536 (16-bit), ranging between 0% and 100% reflectivity. These parameters are the same of two of the main commercial interrogators, the Bayspec FBGA (FBG Analyzer) [36] and the Ibsen I-MON-USB [35], that are the building blocks of most commercial interrogators.

The FBG reflection spectrum has been simulated using Erdogan′s coupled mode theory [3]; the specific details of such well established modeling can be found in [2,3,4] and are also at the basic of the layer-peeling analytic approach [46,47]. On the other side, as FPI sensors are often used in pressure sensors, in their extrinsic version (EFPI) [22,23,24,43], this implementation is hereby used as FPI simulation. The FPI spectrum is generated with the model as in [43], which is also correspondent to the transmission matrix method of Skaar [48,49]. FBG and FPI spectra are generated, and subsequently discretized. Then, white noise is added to the sensor spectrum; in real sensing systems, whereas noise is not Gaussian, a whitening filter can be applied to equalize noise [50].

**Figure 1:**
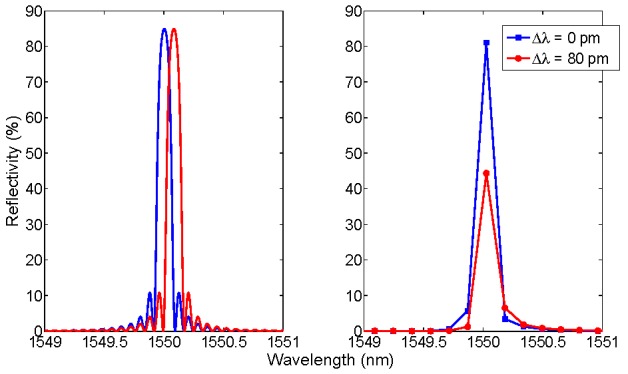
Benchmark for simulation of an FBG sensor. Left: original spectrum, in reference conditions (Δλ = 0 pm) and with a wavelength shift applied (Δλ = 80 pm). Right: reference and shifted spectra after wavelength and amplitude discretization, on a 156-pm wavelength grid.

Figure 1 shows the benchmark for simulation used for FBG sensing. The FBG spectrum is generated at 1550 nm peak wavelength, with 85.0% reflectivity; then, 80 pm wavelength shift is applied. The simulated spectra, in accordance to [3], exhibit a full-width half-maximum (FWHM) bandwidth of 123 pm and side lobes. After discretization, on a 156-pm grid [36,37], the number of useful samples is dramatically reduced as the entire FBG profile is reproduced in four digital samples. It is also possible to notice that the amplitude of the main sample reduces significantly when the 80-pm shift is applied, due to the coarse sampling.

**Figure 2:**
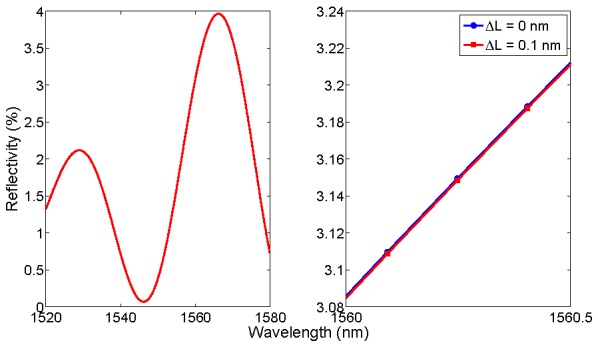
Benchmark for simulation of an FPI sensor. (**Left**) original spectrum, in reference condition (Δ*L* = 0 nm), and with a Fabry-Perot cavity expansion Δ*L* = 0.1 nm; (**Right**) zoom on a 0.5 nm spectral interval.

Figure 2 shows the benchmark for a low-finesse EFPI sensor, based on a glass/air cavity as in [29,30,31,33,43]. The structure is based on an air-gap Fabry-Perot cavity with length *L* = 30 μm, with a glass diaphragm with thickness *d* = 2 μm [32,43]. The resulting spectrum is a convolution of the two cavities, respectively air-gap and glass diaphragm, with a maximum reflectivity around 4%. The original spectrum is then perturbed by changing the air-gap length by a quantity Δ*L*. In Figure 2, spectra are reported with the same 156-pm grid of Figure 1. As a main difference with the FBG, the FPI has a broadband spectral envelope; when the air-gap is compressed or extended, the spectral variations are barely visible, and dependent on the SNR.

The benchmark introduced in Figure 1 and Figure 2 is the main framework for analysis of FBG and FPI sensors. Performances of the KLT-based detection method are evaluated on this benchmark, as a function of the SNR [42]. For FBG sensors, the wavelength shift is linearly dependent upon strain and temperature: for gratings inscribed on glass fibers, typical coefficients are 1 pm/με and 10 pm/°C [2,4,5,6]. For EFPI sensors based on glass diaphragms, the typical pressure sensitivity ranges from −1 nm/kPa [24,29,32,43] to −10 nm/kPa [51], where the negative sign refers to a compression of the cavity. In addition, EFPI sensors are temperature-sensitive, with linear coefficient of approximately 1 nm/°C. Temperature sensors based on intrinsic FPI [52] can be modeled as EFPI sensors with d → ∞.

### 3.2. FBG Interrogation

**Figure 3:**
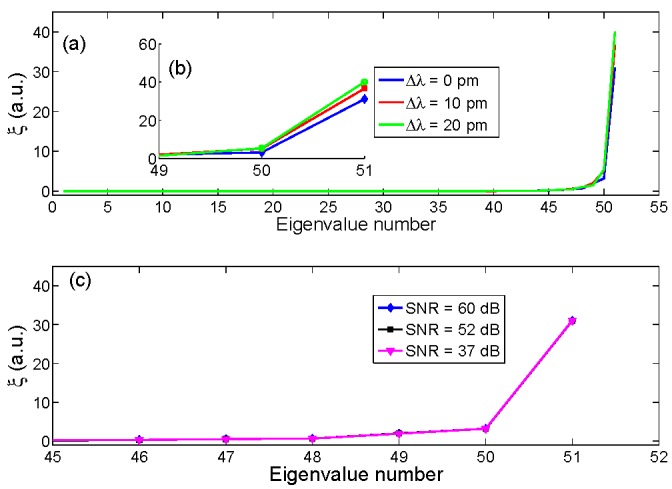
Outline of the KLT-SVD applied to the interrogation of an FBG. (**a**) The eigenvalue string ξ is reported for a grid of *N* = 51, for different values of wavelength shift Δλ = 0 pm, 10 pm, and 20 pm, all having SNR = 60 dB; (**b**) The inset shows the three high-rank eigenvalues from (**a**), highlighting in particular the highest eigenvalue ξ*_N_*; (**c**) For Δλ = 0 pm, the eigenvalue string *ξ* is reported for different values of SNR: 60 dB, as in (**a**), 52 dB, and 37 dB.

The principle of operation of the KLT algorithm applied to FBG interrogation is illustrated in Figure 3. The FBG is sampled on a grid with size *N* = 51, and 156 pm wavelength sampling, with SNR = 60 dB. Figure 3a shows the eigenvalue string ξ for all the 51 eigenvalues. It is possible to divide eigenvalues in three ranks: the low-rank eigenvalues (1–45) have very low amplitude, as they mostly confine the energy of noise; the mid-rank eigenvalues (46–49) combine the energy of the signal with the noise energy; high-rank eigenvalues (50–51) are dominated by the useful signal. In particular, the highest rank eigenvalue ξ*_N_* has amplitude much greater than all other eigenvalues, and it also has the strongest dependence on the wavelength shift. The inset in Figure 3b highlights this dependence, for the three main eigenvalues. The main benefit of the KLT, even compared to FFT-based algorithms as [42], is that it has the capability to neatly separate noise and signal, as demonstrated by Maccone [45,46]. In Figure 3c, for Δλ = 0 pm, the eigenvalue string is reported for different SNR values (60 dB, 52 dB, and 37 dB): it is possible to show that no visible variation of the main eigenvalue can be noticed even when the SNR is lowered.

**Figure 4:**
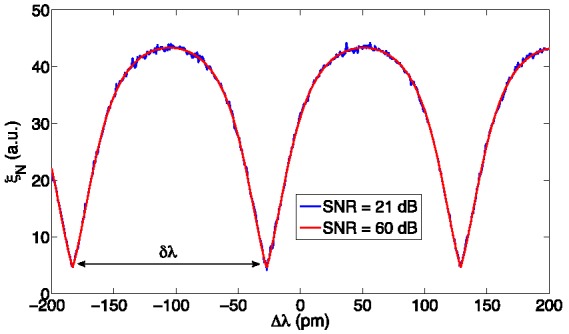
High-rank eigenvalue ξ*_N_* as a function of the FBG wavelength shift Δ*λ*.

Thus, the main driver for FBG interrogation is the estimation of the main eigenvalue ξ*_N_*. Figure 4 reports, for the same benchmark of Figure 1 and Figure 3, the high rank eigenvalue ξ*_N_* as a function of the wavelength shift Δλ, for SNR = 60 dB and SNR = 21 dB. It is possible to notice that the resulting function ξ*_N_*(Δλ) is periodic, with period equal to the wavelength sampling step δλ [47]; thus, the functioning of the KLT algorithm is efficient for small wavelength shift, but for large shifts requires to ballpark the wavelength shift in the correct semiperiod. In addition, it is possible to notice that the function ξ*_N_*(Δλ) provides a good selectivity: around its minima, the function has a steep and almost linear profile, and tends to become quadratic around its maxima. It is possible to notice that in high-SNR conditions, the profile of ξ*_N_* is well defined; noise becomes more relevant in low-SNR conditions (21 dB).

**Figure 5:**
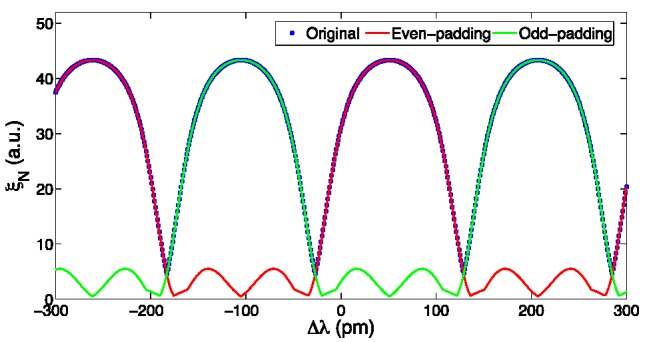
ξ*_N_*(Δλ) function reported for all spectral values as in Figure 4, and padding to zero all even and odd samples input to the FFT in Equation (3).

Hence, the KLT method requires an initial rough estimate of the wavelength shift, in order to select the right semiperiod of the periodic ξ*_N_*(Δλ) function, prior to return the accurate estimation of Δλ. Such a task can be done using standard techniques, such as centroid tracking, Gaussian fitting, or bandwidth tracking [47,53]. However, the KLT provides a way to improve the estimate, through the manipulation of the reflection spectrum *S*[λ]. It is possible to show in Figure 5 that, by zeroing all the even samples of *S*[λ], prior to perform the FFT in Equation (3), a different pattern is obtained. This new function has a period of 2δλ; on the even semiperiods, it is identical to the original ξ*_N_*(Δλ), while on the odds semiperiods it has a different pattern. Conversely, by zeroing the odd samples, the symmetric pattern is obtained, *i.e.*, identical to ξ*_N_*(Δλ) on the odd semiperiods. Thus, it is possible to disambiguate the wavelength shift over a window of 2δλ, as either the left-padded or the right-padded value ξ*_N_* is higher than a threshold (around 6, in Figure 5).

**Figure 6:**
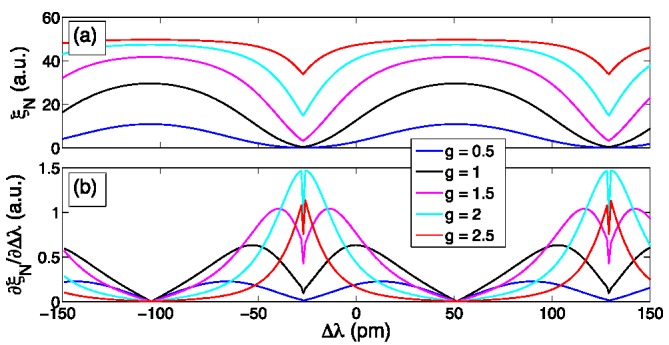
(**a**) ξ*_N_*(Δλ) function for different values of grating strength coefficient *g*; (**b**) derivative of ξ*_N_*(Δλ).

The shape of ξ*_N_*(Δλ) depends on the type of FBG, and Figure 6a shows its dependence on the grating strength coefficient *g*, which is the product of the grating length by the grating coupling coefficient as in [3]; *g* is directly related to the grating reflectivity, equal to tanh^2^(*g*), and to its bandwidth. For small values of *g*, the function ξ*_N_*(Δλ) is similar to a sine wave, whereas it tends to progressively flatten as *g* increases; for *g* = 2.5, the function is almost flat-top. On the other side, larger values of *g* increase the amplitude of ξ_N_. It is then possible to evaluate what is the best value of *g* that returns the best performance. In order to evaluate it, Figure 6b reports the derivative of ξ*_N_* with respect to Δλ. It is possible to show that for *g* = 0.5, the derivative is similar to a sine wave, but its amplitude is small due to the low values of ξ*_N_*; on the opposite, for *g* = 2.5, the derivative is significantly higher than zero only near the transition point. Within the extremes, the values obtained for *g* = 2 are the highest ones near the minima of ξ*_N_*(Δλ) but they tend to fall near its maxima; for *g* = 1 and *g* = 1.5 the trend is opposite. Figure 6 suggest that choosing *g* within 1 and 1.5 is a good solution for tracking bigger wavelength shifts; while for applications requiring exceptional accuracy for small wavelength shift it is more convenient to choose *g* ~ 2 and operate nearby the ξ*_N_*(Δλ) minimum for the top sensitivity.

**Figure 7:**
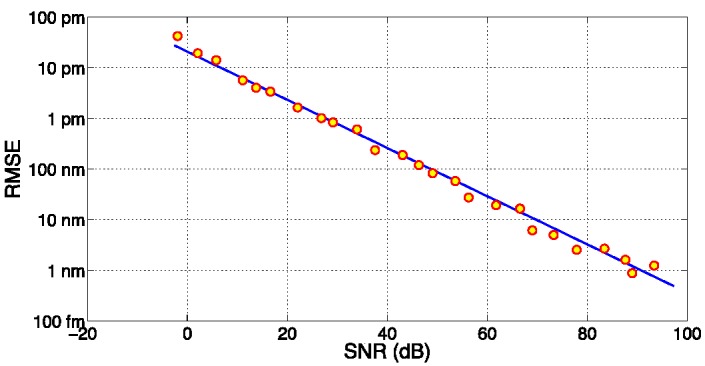
RMSE on FBG wavelength detection, using the KLT-based tracking algorithm, as a function of the SNR; simulation data (dots) and linear fit (line) are reported.

The performances of the KLT-based tracking are illustrated in Figure 7, which reports the root mean square error (RMSE) between the noise-corrupted estimation and reference in absence of noise, as a function of the SNR. Data, reported in logarithmic units, show a good linear trend, and the linear fit shows that RMSE is proportional to 10^−0.0475×SNR[dB]^. The chart shows the benefits of the KLT algorithm: despite a sampling grid of 156 pm, the RMSE = 1 pm threshold is achieved with SNR = 25.3 dB, and the 0.1 pm threshold is achieved for SNR = 48.7 dB, both values still inferior to the SNR of typical interrogation units (>60 dB) [36,37]. These results are comparable with Lamberti′s FFT method [42], but achieved with an inferior sampling grid (156 pm *vs.* 10 pm). Thus, the KLT method applied to low-cost FBG interrogators allows achieving accuracy better than 0.1 με or 0.01°C, despite the coarse sampling.

Results shown in Section 3.2 have been obtained for a grid of *N* = 51 points. The computational complexity of the KLT is O(N^3^). Computation has been carried out on a computer with 2.6 GHz i5 processor, using Matlab software; KLT has been implemented following the approach identified by Maccone [46], with the evaluation of the main eigenvalue. With this setup, the average computation time is 0.998 ms, which is compatible with 1 kHz operation as provided by most static and dynamic interogators [36,37,38].

### 3.3. FPI Interrogation

The interrogation of Fabry-Perot sensors follows the same principle of the FBG interrogation: interrogation is performed on a *N*-size grid, with *N* = 51 to maintain the same speed of operation of FBG detection. The majority of FPI sensors operate with a relatively long air-gap (20–60 μm) and a low-finesse, usually around 0.17 corresponding to a 4% reflectivity between air gap and glass mirrors [20,24,32,43], hence having a broadband pattern similar to a sine wave. In order to interrogate such sensors with a relatively low computational complexity, three simple strategies are possible: (a) sample the spectrum on a short interval, rejecting outer samples; (b) down-sample the spectrum, extending the interrogation bandwidth but increasing the sampling grid δλ; (c) divide the spectrum in blocks, and average the spectrum response on each block. For all such approaches, the working principle is the detection of the highest rank eigenvalue *ξ_N_*, which depends on the Fabry-Perot cavity shift ΔL.

**Figure 8:**
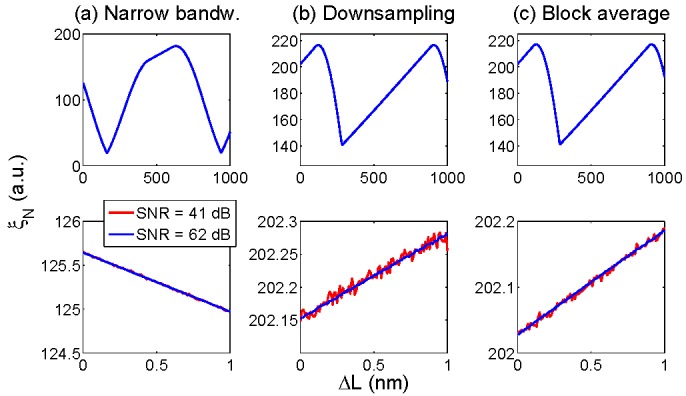
High-rank eigenvalue ξ*_N_* as a function of the Fabry-Perot cavity length variation ΔL. The chart reports the function ξ*_N_*(ΔL) for the three approaches: (**a**) interrogation on a narrow bandwidth (8 nm), with the original sampling grid (156 pm); (**b**) downsampling of spectral response, by a factor 5; (**c**) block-average of spectral response, with block size 5. The upper charts show ξ*_N_* for long values of Δ*L*, up to 1 μm; bottom charts zoom on a variation of ΔL, up to 1 nm. Data are reported for two different values of SNR (41 dB and 62 dB).

Figure 8 outlines the KLT algorithm applies to an FPI sensor. Using a short (8 nm) interrogation bandwidth, the response of the ξ*_N_* function is periodical, with period equal to approximately 740 nm. The envelope is similar to Figure 4, but the function is not symmetrical. Looking at a short variation of Δ*L*, as required by most medical pressure sensors, this approach returns the best resilience to SNR. By down-sampling by a factor of 5, the accuracy for short variations of ΔL worsen, as shown in Figure 8b; conversely, the ξ*_N_* function has a linear pattern for an extended range of ΔL variations, making it attractive for high-temperature sensors [25,54] whereas linearity over broad interrogation range is needed. The block-average, with block size equal to 5, returns a similar response to the downsampling method, but with an improved accuracy as the function *ξ_N_* is more resilient to low SNR.

**Figure 9:**
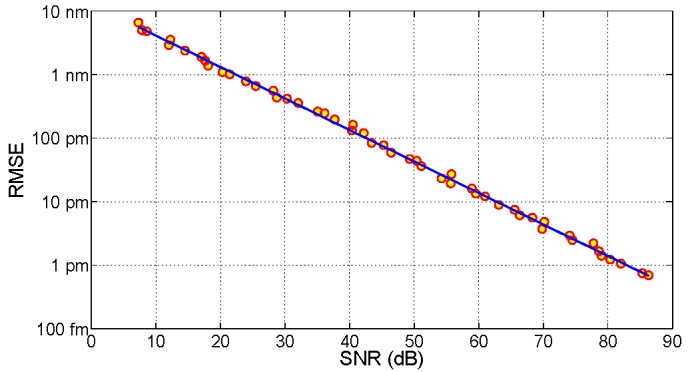
RMSE on FPI cavity length detection, as a function of SNR, using KLT-based algorithm for short-ΔL; the chart shows the estimation (dots) and linear fit (line).

As the main goal of the KLT is to return a high accuracy, the first method is chosen: data are sampled over 8 nm bandwidth, with a 51-size grid. With this setup, by varying the SNR, it is possible to estimate the accuracy on the cavity length Δ*L* detection: accuracy is estimated as the RMSE between the noisy measurement and reference in absence of noise. Figure 9 shows the RMSE as a function of SNR, computed for Δ*L* ranging from 0 nm to 2 nm. Similarly to the FBG tracking, the RMSE is proportional to SNR, in linear units: the linear fit shows that RMSE is proportional to 10^−0.04796^^×SNR[dB]^. As a benchmark, the RMSE is 100 pm for SNR = 42.6 dB, and 10 pm for RMSE = 62.7 dB. With values typical of FBG interrogators and spectrometers optimized for optical sensors [36,37], 1–10 pm RMSE is typically achieved.

The possibility to obtain 1–10 pm accuracy has implications in FPI sensors. EFPI-based pressure sensors fabricated with glass diaphragm have typical sensitivity of 1 nm/kPa; the KLT then allows obtaining accuracy of 1 Pa to 10 Pa. This value corresponds to 0.007–0.07 mmHg or 0.01–0.1 cmH_2_O accuracy in medical units. Considering that low-pressure medical fiber-optic pressure sensors have accuracy of 1 mmHg [55], the KLT allows improving over 10 × to 100 × the accuracy over the state of the art without changing any part of the sensing structure or the interrogator, and despite the relatively low sensitivity, and by just changing the post-processing routine. FPI sensors used as temperature sensors have typical sensitivity close to 1 nm/°C [29]; thus, the KLT allows the detection of the hundredth/thousandth of degree.

## 4. Interrogation of Hybrid FBG/FPI Sensors

**Figure 10:**
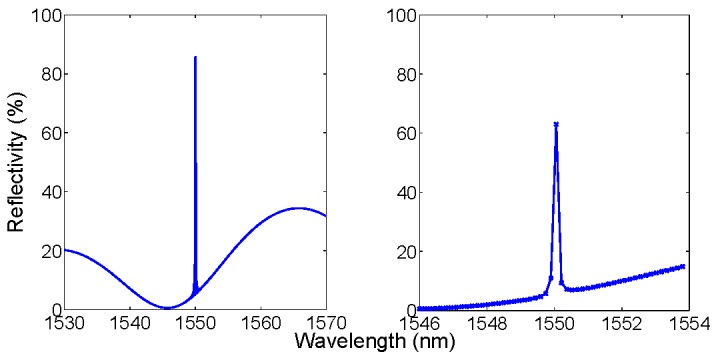
Spectrum of dual EFPI/FBG sensor. (**Left**) original spectrum; (**Right**) spectrum after 156-pm sampling and *N*-size framing.

The KLT can be employed not only to interrogate FBG and FPI sensors, but also as a tool to interrogate hybrid FBG/FPI sensors [31,32,33,34,35]. The spectrum of a dual FBG/FPI sensor has been simulated by combining the FBG in Figure 1 with the FPI in Figure 2. The spectrum of the combined sensor is shown in Figure 10. It results as a combination of FPI and FBG individual spectra, and subjected to both wavelength shifts of the Bragg wavelength and variations of the Fabry-Perot cavity length [35].

**Figure 11:**
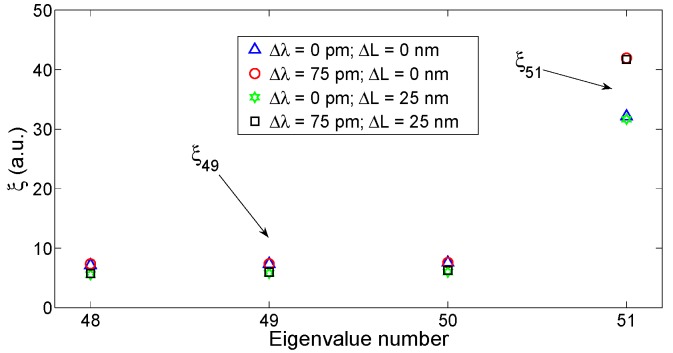
KLT-SVD applied to the dual FBG/FPI sensor. The chart reports the eigenvalues *ξ*, when either the FBG is shifted by 75 pm, or the FPI cavity length is expanded by 25 nm, or both events occur. The chart reports the highest rank eigenvalues (48–51), significant for demodulation.

The results of the KLT eigendecomposition, applied to the FBG/FPI spectrum with different values of Δλ and ΔL, is shown in Figure 11. On one side, it is possible to notice that the main eigenvalue (ξ_51_ = ξ*_N_*) is substantially dependent only on Δλ, and approximately independent on ΔL. When the FBG is shifted by Δλ = 75 pm, ξ*_N_* increases by 10.1; the residual variation due to ΔL is 0.2 (approximately 2%). On the other side, lower rank eigenvalues such as ξ_49_ = ξ*_N_*_−2_ exhibit the opposite pattern: they depend mainly on the FPI cavity length, whereas they are almost independent on Δλ.

This capability of the KLT to separate the FPI from the FBG can be well exploited for dual interrogation. In a first scenario, both the FBG and the FPI sensors are subjected to relatively large variations. This is a common framework in oil and gas and geothermal engineering, whereas the FBG/FPI operates for detection of high temperature (–20°C to >200°C) and high pressure (up to 100 bar) [26,56], as well as in structural engineering, whereas the FBG/FPI structure detects strain and forces [6].

**Figure 12:**
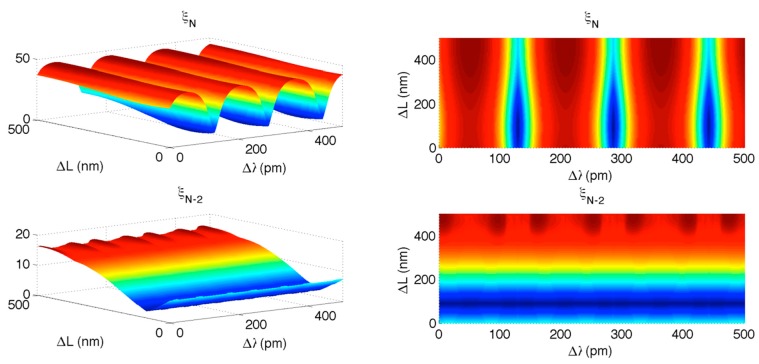
KLT applied to long-range interrogation of FBG/FPI. The upper charts shows the high-rank eigenvalue ξ*_N_* as a function of FBG wavelength shift Δλ and FPI cavity length expansion ΔL; the lower charts show the eigenvalue *ξ_N_*_−2_ as a function of Δλ and ΔL. Left charts plot the full 3D view; right charts show the 2D top-view.

In this condition, it is possible to exploit the separation provided by the KLT. Figure 12 shows, in a 3D chart, the dependence of ξ*_N_* and ξ*_N_*_−2_ on Δ*λ* and Δ*L*. In first instance, it is possible to notice that ξ*_N_* has an almost vertical plot similar to Figure 4, while *ξ_N_*_−2_ is horizontally laid and exhibits a pattern similar to Figure 8. After the preliminary estimate of Δλ and ΔL, the KLT can compute the residual variation: it is possible to estimate ΔL from ξ*_N_*_−2_ chart, and then estimate Δλ from ξ*_N_* as this procedure guarantees a better resilience to the cross-interference.

**Figure 13:**
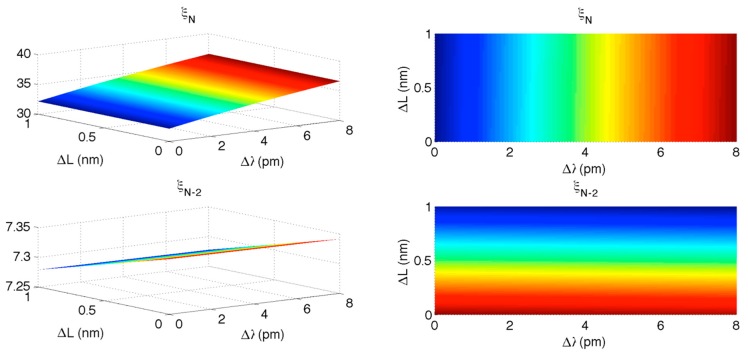
KLT applied to short-range interrogation of FBG/FPI. The upper charts shows the high-rank eigenvalue ξ*_N_* as a function of FBG wavelength shift Δλ and FPI cavity length expansion Δ*L*; the lower charts show the eigenvalue ξ*_N_*_−2_ as a function of Δλ and ΔL. Left charts plot the full 3D view; right charts show the 2D top-view.

In a second scenario, the variations of Δλ and ΔL are confined to small values. This is common in biomedical applications [57], and in this context the benefits of the accurate estimation through KLT are more consistent. Figure 13 shows the KLT output as in Figure 12, whereas the interrogation range has been shortened to 0–8 pm for Δλ and 0–1 nm for ΔL. In this case, it is possible to notice that both ξ*_N_* and ξ*_N_*_−2_ have a nearly planar shape, with limited cross-interference, due to the short range of interrogation. In absence of noise, the error on wavelength estimation Δλ due to variations of ΔL, on a short interrogation range, is estimated as 1.12 pm; the error on ΔL due to variations of Δλ is 0.66 pm. Such uncertainties are then combined with the noise-induced accuracy; for small values of SNR, accuracy is limited by SNR while for higher SNR values, accuracy is limited by Δλ − ΔL cross-interference.

Figure 13 demonstrates that the KLT, without any modification, can interrogate a dual FPI/FBG sensor returning accuracy comparable with the state of the art, and computational complexity comparable with 1 kHz operation, without duplicating the optical source as in [29] or separating FPI and FBG spectra as in [31,32]. It is however possible, increasing the computational complexity, to obtain a neater separation between FBG and FPI spectra; such feature will be described in the next section.

## 5. Interrogation of a Plurality of FBG and FPI Sensors

When a plurality of sensors is encoded in the same spectral window, the demodulation can hardly be performed with classical techniques. On the other hand, the possibility to encode multi-parameter sensors in several active areas along a single fiber is a key feature for optical fiber sensors [58]. Most commercial systems do not enable such features, limiting the interrogation to an array of FBGs, or a single FPI. Dual interrogation, when feasible, is severely limited in accuracy by the scarce possibility of separating the spectral contribution of each sensor to the overall spectrum.

The KLT demonstrates an exceptional capability of separating sensors spectra, encoding their energy into eigenvalues. It is possible then to exploit such principle and reverse the SVD, to facilitate the separation of each sensor. The principle of operation is to manipulate the string of eigenvalues, and consequently modifying the values of ξ by multiplying each eigenvalue *ξ_i_* for a new coefficient *h_i_*:

(7) $$\xi \prime =[h_{1}\xi_{1},h_{2}\xi_{2},\ldots ,h_{N}\xi_{N}]$$

By weighing the eigenvalues, the diagonal matrix $\underline{\underline{D}}$ can be then converted in a new matrix $\underline{\underline{D}}$′ in Equation (5):

(8) $$\underline{\underline{D}}\prime =\left[\begin{array}{cccc} \xi_{1}\prime & & & \\ & \xi_{2}\prime & & \\ & & \ddots & \\ & & & \xi_{N}\prime \end{array}\right]$$

Then, working backwards from Equation (5) to Equation (3), a new spectrum is obtained:

(9) $$\underline{\underline{M}}\prime =\underline{\underline{V}}\times \underline{\underline{D}}\prime \times {\underline{\underline{V}}}^{-1}=\left[\begin{array}{ccccc} G_{1}\prime & G_{2}\prime & G_{3}\prime & \cdots & G_{N}\prime \\ G_{2}\prime & G_{1}\prime & G_{2}\prime & \ddots & \vdots \\ G_{3}\prime & G_{2}\prime & G_{1}\prime & \ddots & G_{3}\prime \\ \vdots & \ddots & \ddots & \ddots & G_{2}\prime \\ G_{N}\prime & \cdots & G_{3}\prime & G_{2}\prime & G_{1}\prime \end{array}\right]$$

(10) $$S\prime \left[\lambda_{1},\ldots ,\lambda_{N}\right]=IFFT\left[G\prime \left(f_{1},\ldots ,f_{N}\right)\right]$$

where the IFFT is the inverse FFT.

Each coefficient 0 < *h_i_* ≤ 1 is a filter on its corresponding eigenvalue. Although it is not possible to set any coefficient to zero, to avoid the determinant of $\underline{\underline{M}}$ to be null, by setting a sufficiently low value of *h_i_* it is possible to “turn off” the *i*-th eigenvalue, and as a consequence the modified spectrum *S*′[*λ*] will not include its contribution. Conversely, by setting *h_i_* = 1, the *i*-th eigenvalue is “turned on”. The coefficient string *h* = [*h*_1_, …, *h_N_*] is then a filter on the eigenvalues, that allows turning on the eigenvalues relevant to a specific type of sensor and turning off the other contributions. In the following, the *i*-th eigenvalue ξ*_i_* is turned on by setting *h_i_* = 1 and is turned off by setting *h_i_* = 10^−8^.

**Figure 14:**
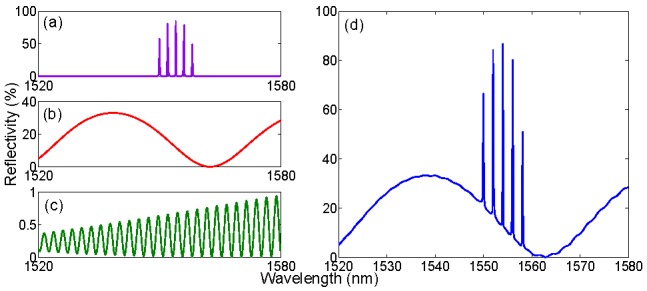
Spectrum of a multiple-FBG/FPI sensing system, used as benchmark for evaluation of the KLT eigenvalue filtering. The system is the combination of three sensing units: (**a**) an array of FBGs, equally spaced; (**b**) a highly reflective FPI with short cavity length; (**c**) a low-finesse FPI with long cavity length. The three spectra are combined, with additive Gaussian noise, to obtain the overall spectrum (**d**).

In order to demonstrate how the eigenvalue filtering operates, Figure 14 presents a relevant benchmark, consisting of the combination of seven sensors. The first batch is an array five FBGs, all having *g* = 1.6 (85% reflectivity), and Bragg wavelength equally spaced by 2 nm starting from 1550 nm; in Figure 14a it is possible to show that, despite each FBG has the same spectral profile, due to the coarse sampling the peak value appears to be different for each FBG. The second sensor is an EFPI, having peak reflectivity 33% and Fabry-Perot cavity length 25 μm; this sensor is a representation of an inline intrinsic FPI (IFPI) sensor fabricated on a splicer with highly reflective mirrors [51]. The last sensor is a lowly reflective EFPI (0.95%), which can simulate pressure sensors based on all-glass structure and low-reflectivity mirrors [57]. The spectrum is acquired on bandwidth of 60 nm with the usual 156 pm sampling; the eigenvalue string length is then *N* = 385.

**Figure 15:**
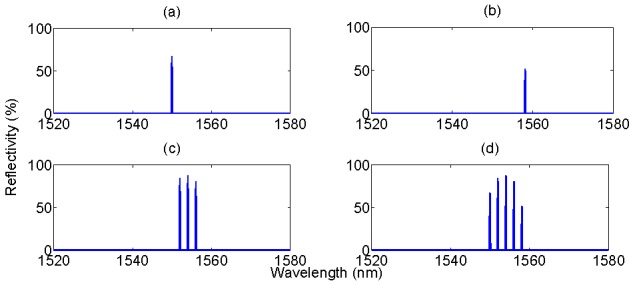
The eigenvalue filtering allows retrieving the FBG profile from the combined spectrum. From the original spectrum in Figure 14d, by means of eigenvalue filtering in Equations (7)–(10), the spectrum of one or multiple FBGs is retrieved. The chart shows four reconstructed spectra, obtained turning on one or more eigenvalues within (*N* − 4) and *N* and turning on all the other ones. The active eigenvalues are: (**a**) ξ*_N_*_−3_; (**b**) ξ*_N_*_−4_; (**c**) ξ*_N_*, ξ*_N_*_−1_, and ξ*_N_*_−2_; (**d**) all eigenvalues from ξ*_N_*_−4_ to ξ*_N_*.

By filtering the eigenvalues following Equations (7)–(10) it is possible to retrieve the contribution of each sensor in the spectrum. In Figure 15, the first part is retrieving the FBGs. It is possible to show that all the five high-rank eigenvalues correspond to the five FBGs that constitute the grating array. In particular, the *N*-th eigenvalue corresponds to the FBG with the highest peak value after sampling, *i.e.*, the third FBG in Figure 14a centered at 1554 nm. The (*N* − 1)-th eigenvalue corresponds to the second FBG (1554 nm), and so until the (*N* − 4)-th eigenvalue that corresponds to the rightmost FBG (1558 nm).

Figure 15 shows some examples of isolating FBGs from the remainder of the spectrum. In first instance, by turning on ξ*_N_*_−3_, and turning off all other eigenvalues, it is possible to retrieve the leftmost FBG centered at 1550 nm. Conversely, the rightmost FBG can be isolated by turning on ξ*_N_*_−4_. It is possible to notice that no trace of any other FBG, or any FPI sensors, appears in the spectrum resulting after the eigenvalue filtering. By turning on more than one eigenvalue, it is possible to isolate more than one FBG. In Figure 15c the three highest rank eigenvalues are turned on: the resulting spectrum shows all the three middle FBGs composing the array, centered at 1552 nm, 1554 nm, and 1556 nm. In Figure 15d all the five high-rank eigenvalues, and the resulting spectrum, contain all the array of Figure 14a, still showing no trace of any FPI spectra. The computation time for one estimation of eigenvalues, filtering, and IFFT, is 101 ms.

By filtering out the eigenvalues corresponding to the FBG peaks, the remainder of the spectrum is encoded in the remaining low-rank eigenvalues. In terms of spectral energy contribution, the IFPI of Figure 14b is the next term that can be extracted. Empirically, this is performed by turning on the eigenvalues ranging from ξ*_N_*_−11_ to ξ*_N_*_−7_, in order to extract those eigenvalues in which the contribution of the other spectral components is lower. Finally, by turning on some low-rank eigenvalues, specifically ξ*_N_*_−240_ to ξ*_N_*_−224_ it is possible to partially recover the spectrum of the EFPI in Figure 14c.

**Figure 16:**
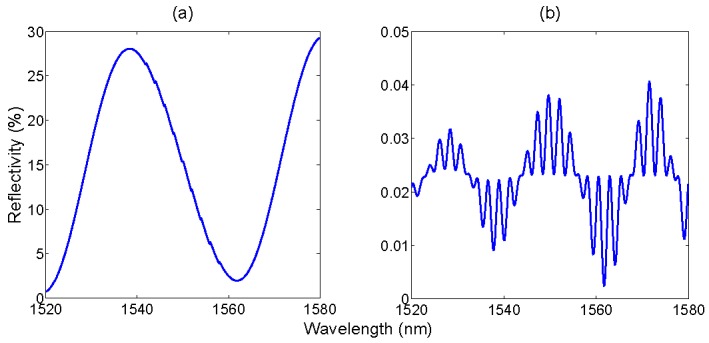
Eigenvalue filtering to retrieve the FPI sensors spectra. (**a**) Spectrum obtained by turning on ξ*_N_*_−11_ to ξ*_N_*_−7_; (**b**) spectrum obtained by turning on ξ*_N_*_−240_ to ξ*_N_*_−224_.

The result of this filtering operation is shown in Figure 16. The second-rank eigenvalues confine most of the highly reflective FPI sensor. By shutting down all components from ξ_1_ to ξ*_N_*_−12_ it is possible to mitigate the effect of noise, the other FPI sensor, and side lobes of FBGs; conversely by removing ξ*_N_*_−6_ to ξ*_N_* it is possible to remove the effect of the FBG peaks. The result is that the spectrum in Figure 16a maintains most of the profile of Figure 14b, with a similar envelope deprived from the FBG array; the peak reflectivity value is lower (27.6%), as a residual part of the energy has been filtered out. In Figure 16b, an empirical reconstruction of the low-reflectivity EFPI has been performed, by switching on ξ*_N_*_−240_ to ξ*_N_*_−224_. In this case, the spectrum results in a different pattern, with a lower reflectivity as part of the energy has been filtered out; however, despite the different background level, the filtered signal maintains the original periodicity, which is a substantial outcome considering that in the overall spectrum, the contribution of this EFPI is barely visible and corrupted by noise. The spectrum in Figure 16b is suitable for estimation of cavity length.

The isolation of each spectral component from the overall spectrum needs to be optimized by a proper choice of the coefficients *h_i_*, and is dependent on the energy associated to each spectral component resulting from the FFT in Equation (3). Individual FBGs, by having a peak spectral profile, or FBG arrays (particularly if equally spaced) are usually confined in the highest rank eigenvalues, and therefore can be well isolated from the background. Low-finesse FPI sensors represent the opposite pattern, as they tend to spread most of their energy in a plurality of eigenvalues. The results obtained in Figure 15 and Figure 16 are obtained with an empirical selection of *h_i_* coefficients, which is suited for the spectrum of Figure 14d; for an arbitrary spectrum, the *h_i_* string needs to be determined. However, due to the nature of the KLT, in a complex spectrum that involves a plurality of sensing elements, FBGs usually to occupy the high-rank eigenvalues, while broadband elements, which spread their energy over a wider bandwidth, are confined to lower-rank eigenvalues.

In this framework, the use of a KLT algorithm can change the framework of lab-in-a-fiber systems based on WDM [58], particularly using a high density of sensors. The typical principle of operation of such systems relies on wavelength separation, by assigning a different wavelength of operation to each sensor. This approach shows vulnerability when broadband sensors such as FPIs, LPGs, or chirped FBGs coexist on the same bandwidth. The KLT, instead, is more capable to disambiguate sensors having different spectral energy, as each of them is encoded in a different portion of the eigenvalue string. The discussion carried out in this section provides a first step in such direction. The future research efforts will focus on optimizing eigenvalue filtering for each type of sensing system; and additional features can be enabled by manipulating the matrix $\underline{\underline{V}}$, by applying a translation matrix in order to improve the isolation of a set of bases from the overall matrix $\underline{\underline{M}}$. After isolating each sensor spectrum, it is possible to reapply the KLT as in Section 3 to accurately track wavelength shifts.

## 6. Experimental Validation

### 6.1. FBG Detection

Experimental validation has been performed with an FBG sensor, and a setup based on white light detection. An optical source, having 1 mW output power and 80 nm bandwidth, has been coupled through a 50/50 coupler, to the FBG sensor. As spectral detector, a Bayspec FBG Analyzer (FBGA) has been used [36], having 80 nm bandwidth, 9-bit (512 points) wavelength sampling, and 16 bit amplitude quantization. The FBG used in experiments has 80.5% reflectivity, 1.2 cm length, and 0.35 nm bandwidth. The FBG has been inserted in a thermoclimatic chamber (Angelantoni Test Chamber CH1200, Angelantoni Group, Cimacolle, Italy), which performs temperature ramps at constant slope.

**Figure 17:**
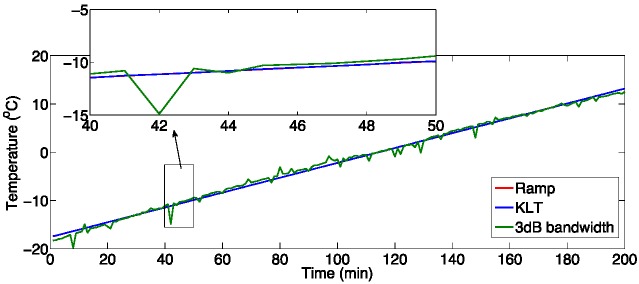
Experimental measurement of temperature with an FBG, in a climatic chamber: the chart compares the reference value, and the temperature values estimated with the 3-dB bandwidth tracking routine, and with the KLT algorithm. The inset shows a smaller portion of the chart, highlighting the better performances of the KLT.

Figure 17 shows the comparison of different tracking techniques, for a temperature ramp applied to the FBG. The reference temperature is acquired from the thermoclimatic chamber, with a PT100 sensor, and is averaged over one minute to reduce uncertainty. In order to simulate a typical FBG tracking method of commercial systems [36,37], the FBG peak has been tracked by measuring the 3-dB bandwidth after spectral resampling. The chart shows that, when the KLT is applied, the uncertainty is significantly lower despite the coarse wavelength sampling. The RMSE in temperature tracking resulting from the KLT is 0.003°C, while the 3-dB bandwidth returns RMSE of 0.1°C.

### 6.2. FPI Detection

Experiments with an EFPI sensor have been performed, in order to validate the performance of KLT tracking; the same optical source and detectors of the previous subsection have been used. The EFPI sensor is an all-glass pressure sensor [43], having sensitivity of 1.6 nm/kPa and air-gap length of 20.1 μm.

**Figure 18:**
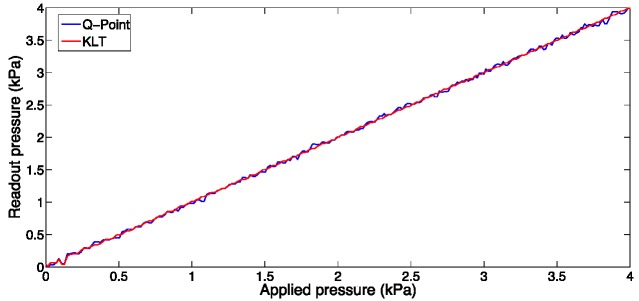
Experimental measurement of pressure with an EFPI, in a reference pressure chamber. The chart compares the pressure detected with Q-point tracking, and the KLT algorithm.

Figure 18 shows the comparison of the KLT algorithm, with Q-point tracking as performed by Bremer *et al*. [29]. Pressure has been recorded in a reference chamber, with accuracy <1 Pa, and set in the range between 0 kPa and 4 kPa. The RMSE between the detected and reference pressure obtained with the KLT is 5.1 Pa, while the RMSE obtained with Q-point is 51.1 kPa.

## 7. Conclusions

The KLT is a powerful algorithm, which applies for detection of signals corrupted by noise, as well as for coarsely sampled signals. It finds excellent application for optical fiber sensors, for the accurate detection of sensors encoded on the same spectral window. Several typologies of sensors and sensing networks can be detected with the KLT, including FBG, and all grating-based sensors, FPI, SPR and other sensors based on absorption. The core of the KLT principle is determining the main eigenvalue that composes the spectral encoding, and thus demodulate the sensor.

The KLT demonstrates a first application in the interrogation of an individual sensor; in this framework, the value proposition of the KLT over other standard methods is that it achieves excellent accuracy, despite a coarse wavelength sampling typical of fiber-optic interrogators based on spectrometers. As a benchmark, FBG and FPI sensors have been simulated, and the KLT demonstrates superior accuracy than the state of the art, overcoming by 10–100 times the limitations of other techniques that struggle with coarse sampling. An experimental validation has been carried out, confirming the performance increase due to the KLT.

A second application that has been outlined is the dual decoding of FBG/FPI sensors: in this case, while the main eigenvalue is substantially dependent on the FBG, the lower rank eigenvalues are approximately dependent on the FPI only. This allows dual demodulation of multi-sensors, with a fast algorithm that does not require duplicating the optical source or the interrogation range.

A final extension of the KLT requires the manipulation of eigenvalues. This principle allows removing spectral components from the overall optical spectrum, thus extracting the spectrum of an individual spectrum and isolating it from the overall spectrum. The KLT shows the premises to operate with dense lab-in-a-fiber sensors, where a plurality of sensors coexist on the same bandwidth and detection is limited by cross-interference. A preliminary demonstration has been carried out, confirming the principle of operation and leaving the margin for potential improvements in wavelength-encoded sensing systems.
